# FGF-7 Dictates Osteocyte Cell Processes Through Beta-Catenin Transduction

**DOI:** 10.1038/s41598-018-33247-8

**Published:** 2018-10-04

**Authors:** Xiao-Yu Liu, Xin Li, Ming-Ru Bai, Xia Chen, Cheng-Lin Wang, Jing Xie, Ling Ye

**Affiliations:** State Key Laboratory of Oral Diseases, West China Hospital of Stomatology, Sichuan University, Chengdu, China

## Abstract

It is well recognized that osteocytes communicate with each other via gap junctions and that connxin43 (Cx43) shows its great potential in gap junction for the contribution enabling transmission of small molecules and operating in an autocrine/a paracrine manner. Fibroblast growth factors (FGFs) play significant roles in new bone formation and adult bone remodeling, and FGF signaling is regulated by the precise spatiotemporal approaches. However, the influence of FGF7 on osteocyte cell processes is not well elucidated. In this study, we aimed to examine the impact of FGF7 on osteocyte cell processes by characterizing the expression of Cx43 and to reveal the underlying mechanism regulating this cell process. We first found that the mRNA level of FGF7 was higher relative to other FGF family members both in osteocytes cell line (MLO-Y4) and bone tissue. We then demonstrated that FGF7 could increase the expression of Cx43 in osteocytes and promote the cell processes in the form of gap junctions between osteocytes. This modulation was due to the FGF7-induced cytoplasmic accumulation and resultant nuclear translocation of β-catenin. Our results could help us to further understand the importance of FGF7 on bone cell behavior and bone physiology and even pathology.

## Introduction

Osteocytes, as one of the key regulators in skeletal system, are the most abundant cell type in bone and are widely distributed throughout the mineralized bone matrix forming an interconnected network that optimally positions them to sense the local and systemic stimuli and resultantly to regulate bone remodeling and adaptation^1^. Indeed, skeletal development and maintenance in post-natal life require extracellular and intracellular autocrine and/or paracrine signaling among bone cells. One of the important ways for the coordination of cellular functions is via the direct cell-to-cell communication via gap junctions^2^. There are 21 connexin genes^3^. Cx43 is the most abundant and most heavily studied protein in all bone cell types including osteocytes, osteoblasts and osteoclasts^4–6^. Additionally, Cx37, Cx45 and Cx46 expression has been detected in osteoblastic cells recently^7^. Each connexin monomer consists of an intracellular N-terminus, four transmembrane domains, two extracellular loops, one cytoplasmic loop, and an intracellular C-terminus^8^. Six connexin subunits can form a ring with a central pore, collectively known as a connexon or hemichannel. An intercellular gap junction or channel is formed when a hemichannel from one cell docks to another hemichannel of an adjacent cell^3^. Gap junctions provide an intercellular pathway for the transmission of ions, nucleotides, small molecules, and second messengers involved in cell-to-cell communication that can contribute to the dynamic and interconnected network of cells^9,10^. And it can initiate the cellular response to mechanical cues, hormonal and growth factor stimulation and regulate complicated skeletal biologic effects^11–14^.

Fibroblast growth factors (FGFs) play an important role in regulating cell proliferation, migration, and differentiation in many organs including bone. FGF/FGFR signaling plays essential roles in the maintenance of bone homeostasis^15^. FGF7, also known as “keratinocyte growth factor”, is a paracrine and/or autocrine mediator. FGF7 binds to its high-affinity receptor FGFR2IIIb and controls various biological processes^16^. Previous studies showed that addition of exogenous FGF7 facilitates osteogenic differentiation of embryonic stem cells through the signal activation of extracellular signal regulated kinase (ERK)/runt-related transcription factor 2 (Runx2)^17,18^. In bone tissue, it was reported that FGF7 showed a positive role in new bone formation by local delivery of FGF7 in mandible defects of rats^19^. However, the roles of FGF7 in osteocyte behavior are not well understood. Previous study characterized the induction of an osteocyte-like phenotype of cultured osteogenic cells upon treatment with FGF2^20^. Since FGF2 and FGF7 are both secreted signaling proteins that signal to the common receptor tyrosine kinases, and furthermore, they are shared by the same core sequence conservation and structure from the point of structural biology^21^, we are interested in whether the profound morphological shift of osteocytes will occur by the induction of FGF7.

In this study, based on the previous reports which showed that nuclear β-catenin activated through the inactivation of glycogen synthase kinase 3 (GSK-3) by PGE2-induced phosphoinositide-3 kinase (PI3K)/Akt and cAMP-PKA signaling stimulates Cx43 expression and gap junction communication between osteocytes^20^.

We aimed to explore the role of FGF7 in osteocyte cell processes and further its potential regulatory mechanisms.

## Materials and Methods

### Reagents

All chemicals were purchased from Sigma-Aldrich Corp, unless otherwise indicated. MLO-Y4 cells (University of Texas, TX, USA). Foetal Bovine Serum (FBS, Gibco, CA, USA). FGF7 (Recombinant Mouse KGF/FGF7, R&D Systems, Minnesota, USA). RNeasy Plus MiniKit (Qiagen, Valencia, CA, USA). DNase I (Thermo Fisher Scientific, Waltham, MA, USA). cDNA synthesis kit (Thermo Fisher Scientific, Waltham, MA, USA). PCR kit (Thermo Fisher Scientific). QuantiTect SYBR Green PCR Kit (Qiagen, Frankfurt, German). TOPO II TA cloning kit (Invitrogen, Carlsbad, CA, USA). Anti-connexin43 antibody (ab11370, Abcam Biotechnology, CA, USA)/Anti-beta-catenin antibody (ab32572, Abcam)/Anti-active beta-catenin antibody (05665, Millipore, MA, USA)/Anti-lef-1 antibody (ab137872, Abcam). Mouse anti-rabbit IgG-HRP (sc-2357 Santa cruz biotech, CA, USA). Fluorescein isothiocyanate (FITC) - conjugated phalloidin (A12379, Invitrogen, CA, USA). 4′6-diamidino-2-phenylindole (DAPI, D8417, Sigma-AldrichSt. Louis, MO, USA). XAV-939 (HY-15147, Selleck, TX, USA).

### Cell culture

The materials used for this study were obtained according to ethical principles, and the protocol was reviewed and approved by our institutional review board (Institutional Review Board at the West China Hospital of Stomatology, No. WCHSIRB-D-2017-029). MLO-Y4 cells were cultured in a 25 cm^2^ flask containing 3–4 ml Dulbecco’s Modified Eagle Medium (high-glucose DMEM, 0.1 mM nonessential amino acids, 4 mM L-glutamine, and 1% penicillin/streptomycin), with 10% fetal bovine serum at 37 °C in a humidified atmosphere of 5% CO_2_ in air.

### RNA isolations and first-strand cDNA synthesis

Osteocytes RNA samples were isolated using the RNeasy Plus MiniKit with a genomic DNA eliminator. Total RNA samples of bone tissue were isolated from the femur and tibia tissue of 4–6 weeks C57BL/6J mice. Isolated RNA samples were dissolved in RNase-free water and quantified by measuring the absorbance at 260 nm with a spectrophotometer. The RNA samples were then treated with DNase I, and cDNA was prepared from each sample, using 0.5 mg of total RNA and the cDNA synthesis kit in a final volume of 20 μl.

### Semi-quantitative PCR

To evaluate the expression levels of FGF members normalized to the glyceraldehyde-3-phosphate dehydrogenase (GAPDH), semi-quantitative polymerase chain reaction (PCR) was performed with a PCR kit, using a thermo-cycler (Bio-Rad, Hercules, CA, USA). Selected sets of primers are shown in Table 1. Basic local alignment search tool (BLAST) was used to search for all primer sequences to ensure gene specificity. Semi-quantitative PCR were performed in a 25 μl volume containing a 1 μl cDNA sample. cDNAs of osteocyte and bone tissue are amplificated in two ways: positive reverse transcription (+RT) and mock reverse transcription (−RT) to eliminate the genomic DNA contamination. The PCR procedure consisted of a 30 s denaturation cycle at 94 °C, a 30 s annealing cycle at 55–65 °C and 72 °C, a 30 s elongation cycle, 25–38 amplification cycles. The products were electrophoresed by 2% agarose gel and visualized by staining with ethidium bromide (EB).

**Table 1:** Primer pairs of all FGFs in mice.

mRNA	Primer pairs
GAPDH	Forward: AGGTTGTCTCCTGCGACTTCA
	Reverse: CCAGGAAATGAGCTTGACAAA
FGF1 (110 bp)	Forward: GCTCGCAGACACCAAATGAG
	Reverse: GAGGCCCACAAACCAGTTCT
FGF2 (162 bp)	Forward: GGCTGCTGGCTTCTAAGTGT
	Reverse: TCTGTCCAGGTCCCGTTTTG
FGF3 (169 bp)	Forward: GACGGCTGTATGCTTCGGAT
	Reverse: CCATTCACCGACACGTACCA
FGF4 (148 bp)	Forward: AAGCTCTTCGGTGTGCCTTT
	Reverse: CTCGGTTCCCCTTCTTGGTC
FGF5 (194 bp)	Forward: GCTGTGTCTCAGGGGATTGT
	Reverse: TACCACTCTCGGCCTGTCTT
FGF6 (152 bp)	Forward: ACACGAGGAGAACCCCTACA
	Reverse: GGAACTTGCATTCGTCGTGG
FGF7 (194 bp)	Forward: CGTGGCAGTTGGAATTGTGG
	Reverse: AGGCAACGAACATTTCCCCT
FGF8 (117 bp)	Forward: GTTGCACTTGCTGGTTCTCTG
	Reverse: AAGGGTCGGTCCTCGTGT
FGF9 (147 bp)	Forward: ACGGTCGGATGGGATGAAGA
	Reverse: TGGCACAGGTTCAAGGTCAA
FGF10 (169 bp)	Forward: GTGCGGAGCTACAATCACCT
	Reverse: CGGCAACAACTCCGATTTCC
FGF11 (142 bp)	Forward: CCAGCTCCTTCACCCACTTC
	Reverse: AAGCGACACTCTGCTGTGAA
FGF12 (95 bp)	Forward: CTCCCACTTCTCGCCTCTTG
	Reverse: AGCTGGGTGAGGTCTACGAA
FGF13 (108 bp)	Forward: GCTAGTCTCGCCTGCCATC
	Reverse: CATGCCTTCTGAGCTCCTCC
FGF14 (127 bp)	Forward:AGCGGCTTGATCCGTCAGAAA
	Reverse: AGAAGATATCCACCAGGTTGCC
FGF15 (132 bp)	Forward: GACTGCGAGGAGGACCAAAA
	Reverse: CAGCCCGTATATCTTGCCGT
FGF16 (166 bp)	Forward: GATCAGCATCAGGGGAGTGG
	Reverse: CTCCGAGTCCGAGTGTTTGT
FGF17 (180 bp)	Forward: CAGTAGCCCAAGAGAGCGAG
	Reverse: TCTCGGAGCCACAGGTTTTC
FGF18 (171 bp)	Forward: GGACCAGTGGGAAGCACATT
	Reverse: CGAGCTTGCCTTTTCGGTTC
FGF20 (179 bp)	Forward: GAGATGGTGCCAGGTCCAAA
	Reverse: TCCTGAACGTCTTCTTCGGTG
FGF21 (148 bp)	Forward: TCTGAACCTGACCCATCCCT
	Reverse: GTCCCAGGGTCCCAACTCTA
FGF22 (128 bp)	Forward: GAGATCCGTTCTGTCCGTGT
	Reverse: TCCCGGAACCTACAGTCCA
FGF23 (144 bp)	Forward: GCAAACGCTCGAACTCTCTC
	Reverse: ACAATCCGAGGTCTCAAGGA

### Quantitative real-time PCR

Quantitative real-time PCR (qPCR) was performed with a QuantiTect SYBR Green PCR Kit using iCycler (Bio-Rad, Hercules, CA, USA) according to operation procedure. qPCR reactions were performed at 0.5 μ mol for each primer in a 25 μl volume containing 1 μl cDNA sample. The reaction was initiated by activating the polymerase with a 5-min pre-incubation at 95 °C. Amplification was achieved with 45 cycles of 15 s denaturation at 94 °C, 15 s annealing at 64 °C and 15 s elongation at 72 °C. The procedure was concluded by the melting curve analysis. All experiments were performed in triplicates. The copy numbers of each gene were determined by cycle threshold (ΔΔCt) methods. Means of the copy numbers of GAPDH were used as internal controls to normalize the data. The standards for establishing standard curves of all primers were prepared from total normal RNA, amplified by qPCR and cloned by TOPO II TA cloning kit, according to the manufacturer’s recommendations.

### FGF7 treatment

Osteocytes were seeded onto six-well plates at 5 × 10^5^ cells per well (85~95%) confluence. Osteocytes allowed to equilibrate for 12 h. Culture media were then replaced with 2% FBS DMEM for a 12 h starvation. Then the osteocytes were divided into the two groups. In the treatment group, the medium was replaced with fresh 1% FBS DMEM containing different concentrations (0.5, 5 and 20 ng/ml) of FGF7. For inhibition experiments, osteocytes were pre-treated with XAV-939 (20 μ mol) for 8 h, then cultured with fresh DMEM containing 20 ng/ml of FGF7 or not. At the protein level, cell lysate samples were collected at 48 h in the control and the treatment groups for western blot.

### Immunofluorescence

Osteocytes were seeded onto 35 mm Glass Bottom Dish and allowed to equilibrate for 12 h. Osteocytes were then cultured for 24 h with fresh 10% FBS DMEM containing either 20 ng/ml FGF7 or PBS solution. After the treatments, the cells were washed 3 times for 2 min and fixed with 4% paraformaldehyde for 10 min, followed by three rinsed with PBS. Then they were permeabilized with 2.5% Triton X-100 for 2 min, washed with PBS. Afterwards, the cells were blocked with 5% bovine serum albumin for 60 min, washed with PBS followed by the addition of anti-connexin43 antibody (1:200)/anti-β-catenin antibody (1:200), and incubated overnight at 4 °C. Following incubation, the cells were washed three times for 15 min with PBS. Goat anti-rabbit IgG and FITC-conjugated phalloidin in PBS were added for double staining, then incubated overnight at 4 °C. Followed incubation, the cells were washed three times for 5 min and then stained by DAPI for nucleus staining. The cell images were captured using a modified confocal laser scanning microscope (CLSM, A1R MP+, Nikon, Tokyo, Japan) and analyzed with Image-Pro Plus Software 6.0.

### Western blot

Protein samples were prepared by mixing one part of sample with one part of Bio-Rad Sample Buffer and then boiled at 100 °C for 5 min. Proteins were separated in 8–12% sodium dodecyl sulfate polyacrylamide gel electrophoresis and transferred to a polyvinylidene difluoride membrane at 200 mA for 2 h in ice treatment. The blot was blocked with 5% fat-free dry milk suspended in 1× TBST for 15 min at RT. Resulting blot was incubated with antibodies (1:500–1:1000), including connexin43/β-catenin/active β-catenin/lef-1 for overnight at 4 °C, followed by incubation with 1:2000 anti-IgG-HRP (AlexSeries, Abcam) for 2 h at RT. Signals from the blots were obtained using a Western Blotting Luminol Reagent Kit.

### Bioinformatics

The information about Genebank ID, gene sequences, the promoter sequences were all from NCBI resources (https://www.ncbi.nlm.nih.gov/) and BioGPS (http://biogps.org/#goto=welcome). The binding site location was achieved through online tool - PROMO (http://alggen.lsi.upc.es/cgi-bin/promo_v3/promo/promoinit.cgi?dirDB=TF_8.3) as previously described^22^. The detailed binding site information was supplemented in Supplementary Material-1.

### Statistical analysis

All experiments were performed in triplicate and reproduced at least three separate times. Statistical analysis of the data was performed with SPSS 16.0 using independent sample t-test analysis to determine whether differences existed. The critical significance level was set to p < 0.05.

## Results

### mRNA level of FGF7 is higher compared with other members of FGF family both *in vitro* cell line and *in vivo* bone tissue

**Figure 1:**
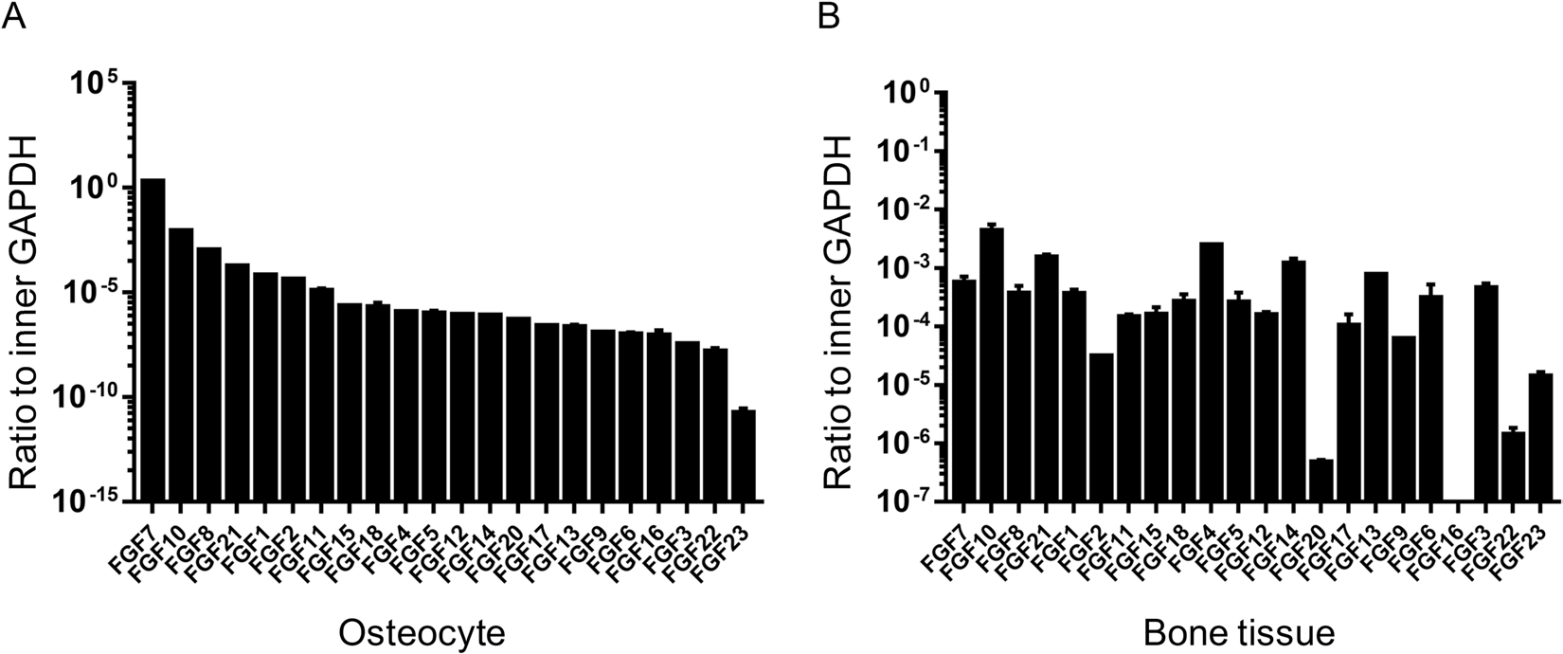
Differential mRNA levels of FGF family in osteocytes and bone tissues. (**A**) The mRNA level of FGFs in MLOY4 cell line. Quantitative real-time RT-PCR analysis was performed on RNA extracted from MLOY4 to determine transcript copy numbers of FGFs relative to the reference gene GAPDH. The indicated values are ratios of copy numbers of each gene. Data were means of three independent experiments (n = 3); bars ± SD. (**B**) The mRNA level of FGFs in bone tissues. Quantitative real-time RT-PCR analysis was performed on RNA extracted from femur and tibia of C57BL/6J mice to determine transcript copy numbers of FGFs relative to the reference gene GAPDH. The indicated values are ratios of copy numbers of each gene. The data were means of three independent experiments (n = 3); bars ± SD.

**Figure 2:**
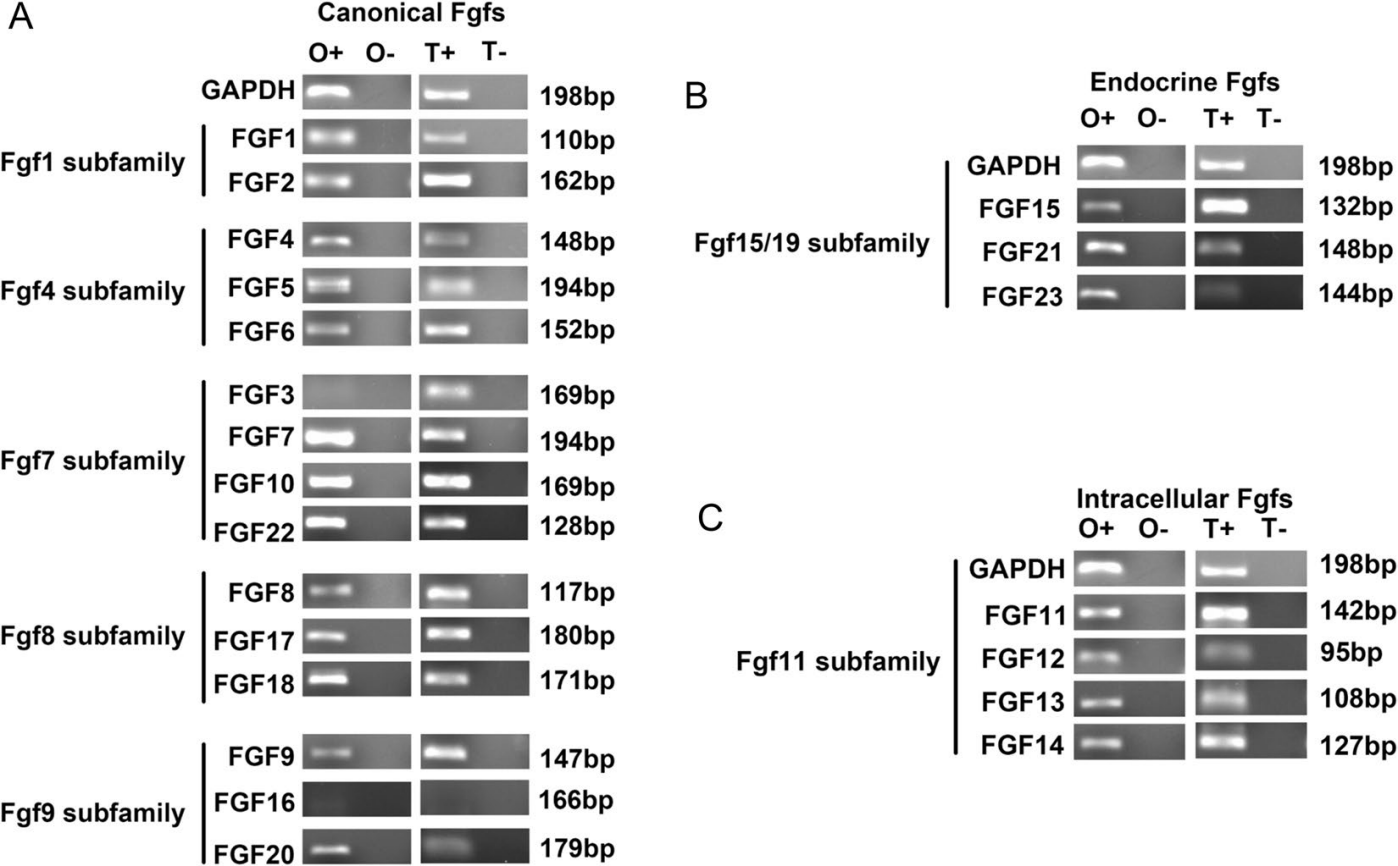
The relative expressions of FGF family showing the mRNA changes in osteocytes in comparison to long bone tissues. (**A**) The comparative expressions of canonical FGFs in both osteocyte cell line and bone tissue. (**B**) The comparative expressions of endocrine FGFs in both osteocyte cell line and bone tissue. (**C**) The comparative expressions of intracellular FGFs in both osteocyte cell line and bone tissue. The results shown were performed by reversed transcript PCR and revealed by relevant regions of ethidium bromide (EB) - stained agarose gels (2%). O+ and T+ represent reverse transcribed RNA; O− and T− represent mock reversed transcribed RNA. Size (in bp) of PCR products are indicated to the right of each panel. The data indicated were means of three independent experiments (n = 3).

We screened twenty-two family members of FGFs (FGF1-23, wherein FGF15 is the mouse ortholog of human FGF19) which have been identified in C57BL/6J mice (Figs 1 and 2). In order to establish methods for detecting mRNAs encoding known FGFs, the primer pairs of the entire FGF family members in mice were designed (as listed in Table 1). Here, we first determined the expression of the mRNA levels of the entire FGF family in osteocyte cell line MLO-Y4 cells with 22 unique PCR primer pairs (Figs 1 and 2). Using quantitative real time PCR, the transcripts of the whole FGF family members were detected (Fig. 1A). Resultantly, there was a significant expression of mRNA level of FGF7 *in vitro* osteocytes. We then used RNA samples isolated from the femur and tibia tissue of 4–6 weeks C57BL/6J mice to confirm mRNA levels of the entire FGF family. We found that the FGF7 gene showed a relative lower mRNA level in bone tissue than in osteocyte cell line but still exhibited a higher level compared with other members of the entire FGF family (Fig. 1B). This broad array of expressed FGFs reflects the fetal stage of the bone tissue. FGFs can be divided into three subfamilies: canonical, hormone-like, and intracellular. Using semi-quantitative PCR, compared with quantitative real time PCR, we demonstrated that among the entire FGF family, FGF7 and 10, were the predominant FGF members expressed both in osteocyte cell line and bone tissue, However, FGF16 and 23, were weakly detectable (Fig. 2A–C). Taken together, we identified FGF7 which was predominantly expressed in osteocyte cell line and bone tissue and thus may serve as the autocrine or paracrine physiologic ligand for FGF receptors.

### FGF7 changes the morphology of the osteocytes and induces a dose-dependent increase of the connexin43 *in vitro*

**Figure 3:**
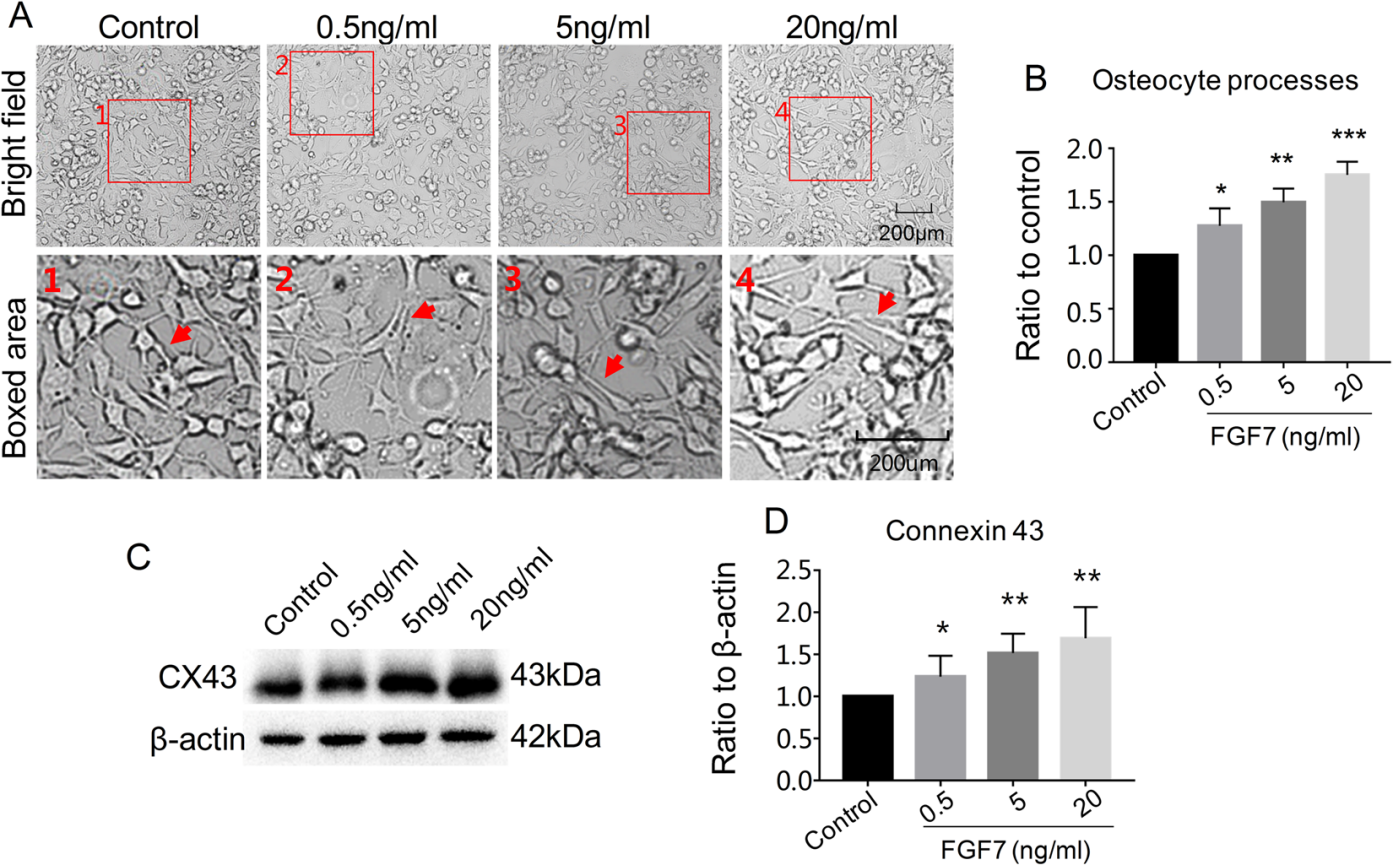
FGF7 induced the morphology of the osteocytes to a more stellate shape, increased the expression of connexin43 in a concentration-dose dependent manner. (**A**) Cell morphologies after FGF7 induction at the concentration of 0.5, 5, 20 ng/ml for 24 h and the red arrows showed the increased osteocyte cell processes in the higher magnification of the boxed area. (**B**) Quantitative analysis of the number of osteocyte processes of (**A**) with Image-J software 6.0. Data were means of three independent experiments (n = 3); Significant difference with respect to control, *P < 0.05, **P < 0.025, ***P < 0.001. (**C**) Western blots showed connexin43 expressions after FGF7 induction increased in a concentration-dose (0.5, 5, 20 ng/ml) dependent manner. The cell lysates were collected at 48 h following FGF7 treatments for connexin43 and beta-actin; the images were collected from two gels with the same loading amounts. The blot gels shown are representatives of three independent experiments (n = 3). (**D**) Quantitative analysis of western blots of (**C**) with Image-J software 6.0. Data were means of three independent experiments (n = 3); Significant difference with respect to control. *P < 0.05, **P < 0.025.

We next focused on the FGF7, the highest expressed in both osteocyte cell line and bone tissue, explored its role in bone function. By using the recombined FGF7, we found that FGF7 could modulate the morphology of the osteocytes in the form of changes of osteocyte cell processes. Briefly, as we can see in the bright field and more apparently in the magnified boxed area, within the effect of different concentrations of FGF7 (0.5, 5 and 20 ng/ml), one of the first change detected in the osteocytes was the formation and elongation of the cell processes (Fig. 3A). And the quantification further showed the number of osteocyte processes increased. (Fig. 3B). Furthermore, after treatments of FGF7 from 0 (control), 0.5, 5, to 20 ng/ml for 48 h, the expression of Cx43, which is regarded as a protein marker of gap junction, was increased in a dose-dependent manner by western blot (Fig. 3C), and the quantification further confirm the increase (1.23, 1.52, and 1.69-fold) with the increased concentrations of FGF7, respectively (Fig. 3D).

### FGF7 regulates CX43 expression and promotes the elongation of osteocyte cell processes

**Figure 4:**
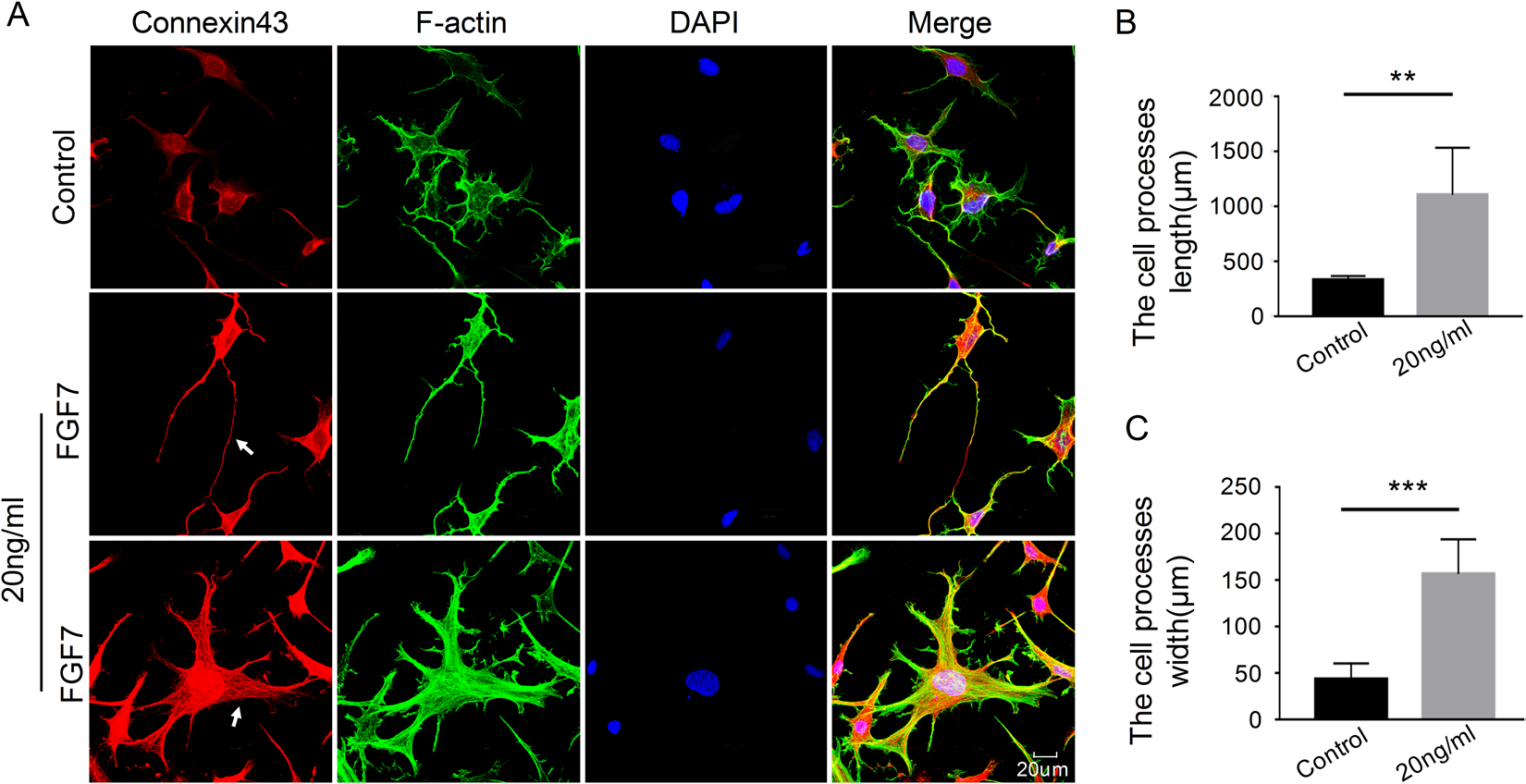
Expression of connexin43 increased after the treatment of FGF7. (**A**) Immunofluorescent stain showed the increased expression of connexin43 in osteocytes at 24 h after the treatment with FGF7 (20 ng/ml). The middle lane with FGF induction indicated the elongation of osteocyte cell processes and the bottom lane with FGF induction indicated the width of osteocyte cell processes. Images shown were representative of three independent experiments (n = 3). (**B**) Different lengths of the osteocyte cell processes in examples of the control and the treatment group. Lengths of the cell processes were analyzed using Image-J software 6.0. Data expressed were means of three different experiments (n = 3). (**C**) Different widths of the osteocyte cell processes in examples of the control and the treatment group. Widths of the cell processes were analyzed using Image-J software 6.0. Data expressed were means of three independent experiments (n = 3); Significant difference with respect to control. **p < 0.025; ***p < 0.001.

**Figure 5:**
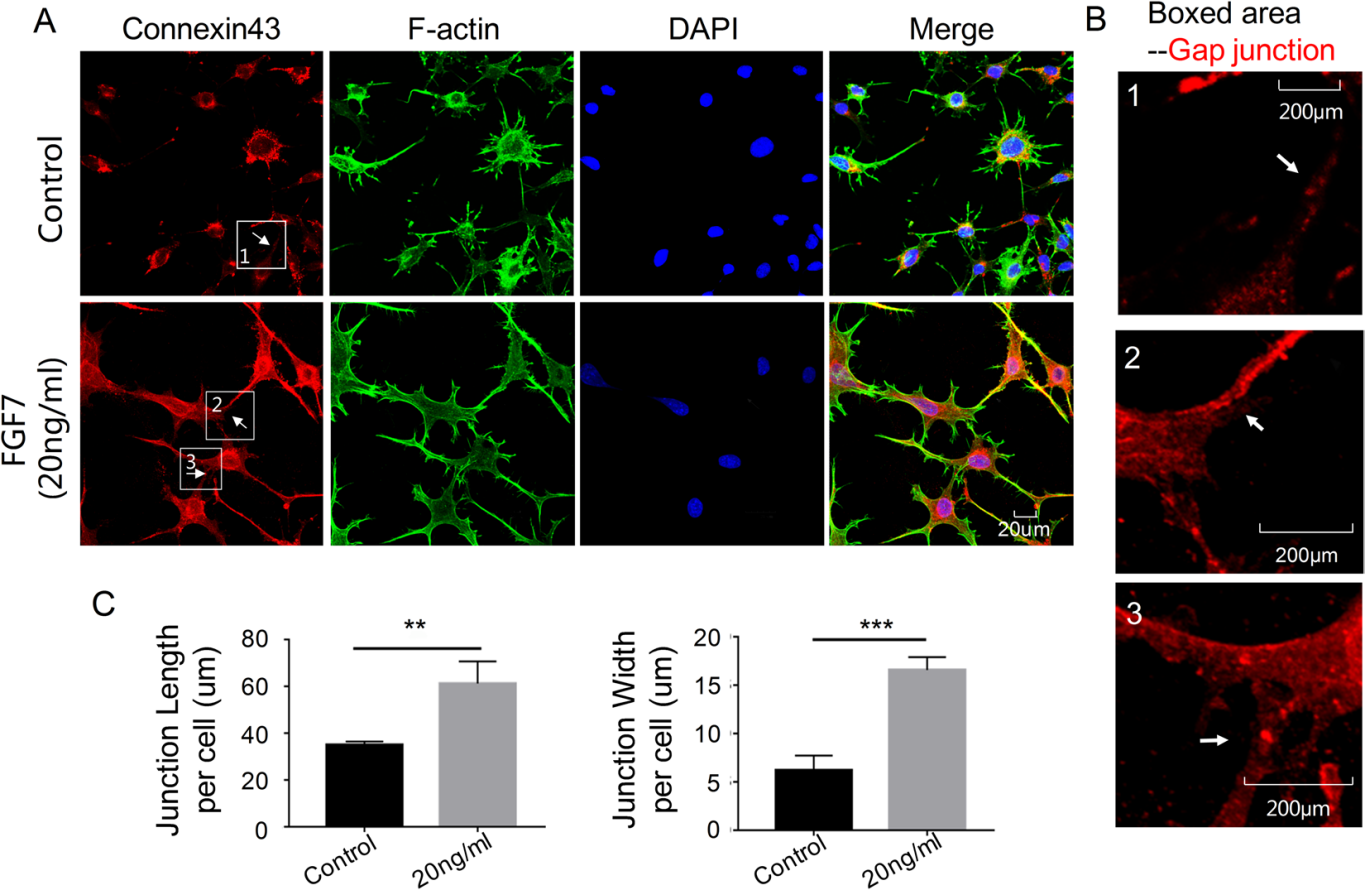
The elongation of gap junction after the treatment of FGF7. (**A**) Immunofluorescent stain showed the elongation of gap junction between osteocytes at 24 h after the treatment with the FGF7 (20 ng/ml). Images shown were representative of three different experiments (n = 3). (**B**) The higher magnification of boxed area showed the elongation of gap junction, as the white arrow indicated. (**C**) Different lengths and widths of the gap junction per cell in examples of the control and the treatment group. The quantitative data were analyzed using Image-J software 6.0. Data expressed were means of three independent experiments (n = 3). Significant difference with respect to control. **p < 0.025; ***p < 0.001.

In order to further indicate the distribution change of Cx43 in osteocytes after FGF7 induction, we used CLSM to confirm its variation. Briefly, osteocytes were cultured with FGF7 at concentration 20 ng/ml. After 24 h of *in vitro* induction, we found FGF7 increased Cx43 and further modulated cell processes compared with the control group (Fig. 4A). The diameter of lengths (Fig. 4B) and widths (Fig. 4C) of the cell processes were increased to 334%, 360%, respectively. To explore whether FGF7 plays a role on the formation of the gap junction, we cultured the osteocytes with FGF7 (20 ng/ml) as we previously described. After 24 h of *in vitro* induction, we found FGF7 promoted the new gap-junction formation compared with the control group (Fig. 5A), and more apparently in the magnified boxed area (Fig. 5B). The quantification further showed the length and width of per cell gap junction were increased to 175% and 267%, respectively (Fig. 5C). Herein we proved that FGF7 signaled in a paracrine manner on osteocytes to modulate cell processes.

### FGF7 modulates osteocyte cell processes through beta-catenin transduction

**Figure 6:**
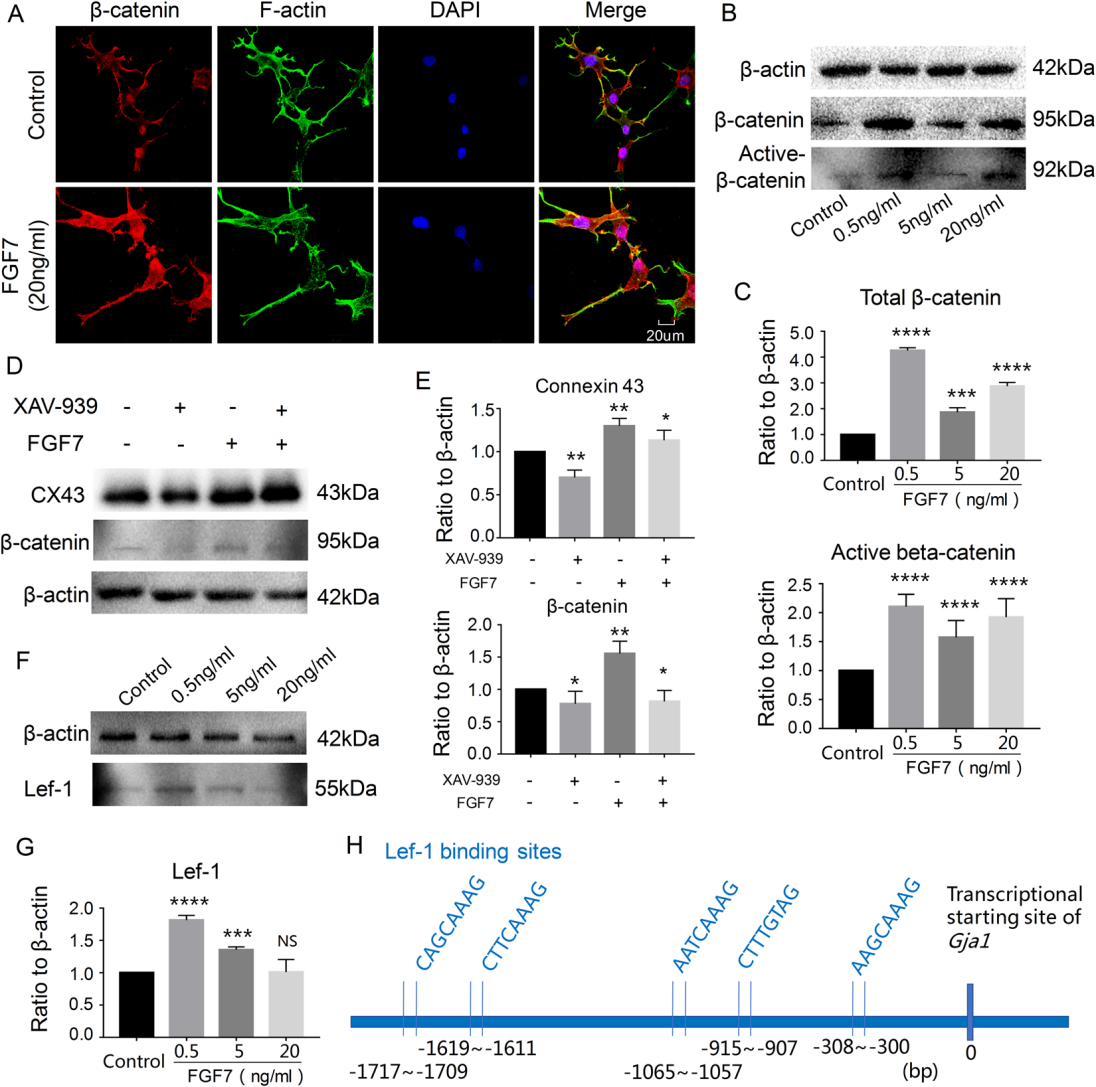
The gap junction elongated through beta-catenin transduction. (**A**) Immunofluorescent stain showed the nuclear accumulations of beta-catenin in osteocytes at 24 h after treatment with FGF7 (20 ng/ml). The images shown are representative of three different experiments (n = 3). (**B**) Western blot showed beta-catenin and active beta-catenin expressions after FGF7 induction increased in a concentration-dose (0.5, 5 and 20 ng/ml) dependent manner. Cell lysates were collected at 48 h following FGF7 treatments for beta-catenin, active beta-catenin and beta-actin; the images were collected from two gels with the same loading amounts. The blot gels shown are representatives of three different experiments (n = 3). (**C**) Quantitative analysis of western blots of (**B**) with Image-J software 6.0. The data shown are representative of three independent experiments (n = 3). ***P < 0.001, ****p < 0.0001. (**D**) Western blot showed the inhibitor of β-catenin, XAV-939, reduced the Cx43 level. In the presence of FGF7, β-catenin inhibition partially reduced the Cx43 in relative to FGF7 induction group. The blot gels shown are representatives of three different experiments (n = 3). (**E**) Quantitative analysis of western blots (**D**) with Image-J software 6.0. The data shown are representative of three independent experiments (n = 3). *P < 0.05, **P < 0.025. (**F**) Western blot showed Lef-1 expressions after FGF7 induction increased in a concentration-dose (0.5, 5 and 20 ng/ml) dependent manner. (**G**) Quantitative analysis of western blots of (**F**) with Image-J software 6.0. The data shown are representative of three independent experiments (n = 3). ***P < 0.001, ****p < 0.0001, NS, no significant difference. (**H**) The bioinformatics showed that Lef1 has the binding sites in the promoter of *Gja1*, the gene name of Cx43.

To explore the possible downstream effectors of FGF7-FGFRIIIb activated signaling involved in the regulation of Cx43 expression, the effect of FGF7 on beta-catenin signaling and its downstream Lef-1 were examined. We found that the expression of both beta-catenin and active beta-catenin were increased (Fig. 6B,C). After the treatments of FGF7 (20 ng/ml) for 24 h *in vitro* induction, immunofluorescence also confirmed the accumulation of cytoplasmic beta-catenin and partial nuclear translocation (Fig. 6A). As the Cx43 serves as a potential target of beta-catenin signaling^23^, we deduced that the Wnt signaling pathway can regulate Cx43 expression to some extent. And then we used the inhibitor of β-catenin, XAV-939, to inhibit the β-catenin expressions and found that β-catenin inhibition reduced the Cx43 level. In the presence of FGF7, β-catenin inhibition partially reduced the Cx43 in relative to FGF7 induction group (Fig. 6D,E). Furthermore, after treatments of FGF7 from 0 (control), 0.5, 5, to 20 ng/ml for 48 h, the expression of Lef-1, the downstream protein of β-catenin, was increased by western blot (Fig. 6F,G). We finally found that Lef1 has the binding sites in the promoter of *Gja1*, the gene name of Cx43, by bioinformatics (Fig. 6H). This provides the potential and direct role of β-catenin in modulating gap junction formation.

## Discussion

Osteocytes occupy the lacunar space reside within the mineralized bone matrix and send cell processes through tiny tunnels called canaliculi to form a canalicular network. Of the major cell types in the bone, osteocytes remained unknown for a long time and defined primarily by their morphology and location rather than by their function^24^. In fact, osteocytes have an extensive network through long, dendritic-like cell processes. Numerous dendritic processes connect osteocytes with adjacent osteocytes and with osteogenic cells on the bone surface via connexin43 (Cx43)-containing gap junctions, the result is a functional syncytium of cells throughout the bone^25^. Abundant data suggest that Cx43 and gap-junction contribute to osteoblastic proliferation and differentiation *in vitro*^26–28^. Meanwhile, *in vivo* studies, Cx43 null mice display impaired intramembranous bone formation also strongly show that Cx43 may be involved in osteoblastic signaling processes^29,30^.

Numerous studies have shown that canonical FGFs, such as FGF2, act in bone. Compared with FGF2, other canonical FGFs have not be studied in detail. Meanwhile, previous study showed that treatment of human skin with recombinant KGF leads to the expression of fibrotic markers^31^. The expression of FGF7 but not of FGF6 and FGF8 is detected in primary osteoblast cell model^12^. Previous study identified the interaction of FGF7 with FGFR2IIIb^32^.

The Wnt signaling pathway has been highlighted in the regulation of bone homeostasis^33^. Beta-catenin is the obligatory transducer for canonical Wnt signaling which is reported to accumulate in the nucleus and bind transcription factors of the high-mobility-group (HMG) box Tcf/Lef family and then stimulates downstream gene expression^34,35^. Further, recent studies identified that osteocytes, as central mediators, control the canonical Wnt signaling pathway in bone and thus induce bone anabolism^36^. Previous study has been demonstrated that Cx43 itself is a target of Wnt signaling pathway^37^. The association of beta-catenin with the 5′ promoter region of Cx43 in MLO-Y4 cells was determined by CHIP assay, and moreover the association of the Cx43 promoter with Pol II and LEF1 was demonstrated^23^.

In this study, we found the following: (1) the mRNA level of FGF7 is higher when compared with other members of FGF family both *in vitro* and *in vivo*. (2) FGF7 can increase Cx43 expression and promote gap junction elongate, likely independent of intracellular signaling pathways that may involve concomitant FGF7 induced the accumulation of cytoplasmic beta-catenin and partial nuclear translocation.

There are limitations in this study. First, although MLO-Y4 cell line have proved to be a very useful tool for studying osteocytes *in vitro*, they cannot provide information on the temporal changes in gene expression and morphology which occur as an osteoblast differentiates into an osteocyte^24^. As osteocytes are gaining increasing interest as a target of therapeutics to increase bone mass. We hope to further study the effects of FGF7 on primary osteocytes. Second, as far as we know, FGF7 deficient mice developed a somewhat greasy or matted appearance in the hair coat, especially among the male mutant mice. Moreover, FGF7 is not required for the development of many mesenchymal epithelial organs, including salivary glands, kidney, lung, spleen, liver, small intestine, and heart^38^. Later, some studies demonstrated that FGF-7 levels modulate the extent of ureteric bud growth and the number of nephrons in the kidney^39^. And others suggest that FGF7 deficiency impairs inhibitory synapse formation, which results in mossy fiber sprouting and enhanced neurogenesis^40^. At present, there are still lacking studies examining whether deletion of the Fgf7 gene impacts on bone formation and pathology, or the phenotype of FGF deficiency mice may be not very significant due to multiple complicated factors involving in this modulating process. However, in the current study, we show changes of Cx43 in osteocytes in response to recombined Fgf7. This result could provide evidence for the important role of Fgf7 in osteocyte behavior. Third, the intracellular effects of FGF7 activated via complicated signal transduction pathways, we cannot eliminate the involvement of other signaling mechanisms. Many signaling pathways combined with other uncharacterized pathways ultimately lead to the increased expression of Cx43 and the formation of numerous functional gap junction channels to accommodate the effects of FGF7. Fourth, Previous study showed that ERK activity is required for the full effect of Cx43 on osteoblast responsiveness to FGF2^41^. And others suggest that Runx2 is a down-stream transcription factor of ERK signaling involved in FGF7-mediated facilitation of DAG-induced differentiation in mouse ESCs^17^. Furthermore, FGF7 treatment augments mineralization of rat BMSCs with increased expression of osteogenic marker genes and this augmentation is suppressed by inhibitors of JNK and ERK^19^. However, in the current study, we only focused on β-catenin signaling but not elucidated the mechanism of MAPK signaling pathway. It should be investigated in the next work.

Further studies need to clarify the concrete molecular mechanisms between Cx43, beta-catenin, and skeletal homeostasis. More detailed experiments using FGF7 and various osteoblastic cells will be needed to further confirm its exact role in bone formation. Last but not the least, we hope that we can explore bone cell behavior and bone physiology or pathology through an eye of the FGF7 in the future.

## Electronic supplementary material

Supplementary Material
