# Supplementary material for: FGF-7 Dictates Osteocyte Cell Processes Through Beta-Catenin Transduction
Source: Sci Rep. 2018 Oct 4;8:14792. doi: 10.1038/s41598-018-33247-8 (PMC6172271; doi:10.1038/s41598-018-33247-8)
Supplement: Supplementary file 1 — Supplementary Material [file 41598_2018_33247_MOESM1_ESM.docx]

*Original article*

**FGF-7 dictates osteocyte cell processes through beta-catenin transduction**

**Xiao-Yu Liu ^a^, Xin Li^a^, Ming-Ru Bai ^a^, Xia Chen ^a^, Cheng-Lin Wang ^a^, Jing Xie ^a*^, Ling Ye ^a,*^**

^a^State Key Laboratory of Oral Diseases, West China Hospital of Stomatology, Sichuan University, Chengdu, CHINA

**Supplementary material - 1.**

The detailed information of binding sites at the promoter of CX43 (*Gja1* gene)

**Connexin 43**

The sequences in capitals at the promoter of CX43 (Gene ID: 12393, Chromosome 17 - NC_000083.6, -2000bp in capitals ~ +200bp in grey (SST))

Binding site 1: AATCAAAG – Dissimilarity: 0.53%;

Binding site 2: AAGCAAAG – Dissimilarity: 1.84%;

Binding site 3: CAGCAAAG – Dissimilarity: 1.84%;

Binding site 4: CTTCAAAG – Dissimilarity: 6.71%;

Binding site 5: CTTTGTAG – Dissimilarity: 7.08%;

ATAGAACTGTCTGGATATGGGCTCCAAAGTTTTTCCTTGTTTGCTTGACCCTTGTGGACTACCCTTCACTCCTAGTCCAGTATTTTCTGCTAAATTAGATAGAGGCTCTTTCTTTTATGTGCTCGAACCATTACACTGTCCATCAAGATTATCCCCTTCTCCTCAAGCCTGGCTGTTTAACTCTGGACCGTGCCAGACTTCACTCCTCTCTGTTTACACGTCATATACTAATGTCACCTTCTGAATTGTCATTTCACAGTCTGAAATACTTCCCAGAAAGAAGCAGCAAAGTCCTTTGTCTACAACTTTTTAGAAAATACTATTTGCGCGGGCCTGCCCACCCACATCTTACTATTCTAGTTTTGGCAGGAATCTAGCCATCTTCAAAGGCACCCGTGGAGTGGGGAGAGAGGTTCCCAACCCCACCCCTCCCTCAGGAGCTATAGGCAGTTCATTATTGGATCTTTGACTACAACTTTTCTGAAAGGCTCTGCTGAAAAGAAACAGCCTCTGTGTCTGCTACTTCTTGCTTTGACTCTGATTACAGAGCTTAACTTTGCAGCCCTTGTCGGCCTGGAACATTGTGTAGACCAGGCTAGCCTCATGAGCATAGTGATTCTCTTCTTGTTTCTACATGCTAAGTGCAGAAACTATAGGCATGAGGTTCCAGCCCTGGCTAGGTGAAAGTGATTGCACTGGCTCCAGGCCCAAAGTTCAGTAAAGAACTGTTAAAATGCTAAGTACCACAAGATGAAAAGCTTTTTTTTTTTTTTTTTTTTGACAAATGCCTACCCCGTCTCTCTGTTGTTGTTTTGCATGTTACTTGATGTTTTAATTTTATTCTGTATCGCCTTGCCTGTCCTGGAACTCACTCTGTAGACCAGTCTGGCCTTTAATTCAGAAATCCGCCTGCCTCTGCCTCTCGAGTGCTAGAGAATCAAAGGTGTGTGCCACACCACCAGGCTCAGAAAATACTCTAATGTGGTCCCTTTTTGTTTATCAAGCTGTTAAGTAACAGTGAGACTTTTTTTTTTTTTTTTTTTAGACAGGGTCTCTCTGTATAGCCCTGGCTGTCCTGGAACTCACTTTGTAGACCACGCTGGCTTCGGACTCAGAAATCCGCCTGCCTCTGCCTCCTGAGTGCTCGGATTATAGGCGTGCACCATCATGCCCGGCACAAGTGAGACAAATTGAACTCAGTTGAAATGTGTACATTAGTCTGAAGATCTTATGTAGGCCGACTTTCAGGGTAGCCTCTGTCTCACAGCTCTAATCCTCCTCCCTCATGCTAGGAAAGTATGAGAACACATGCCTATAATCTCAGAAGGCAAAGATGAGAAGATAACATGTCAGGCTAATATAGGAGACCCATGAAAAAGAAAGAAAGAAAGAAAGAAAGAAAGAAAGAAAGAAAGAAAGAAAGAAAGAAAGAAAGAAAGAAAGGGTTCAGATGAACTTAATTCAGAATGTGCACAAATTACATGTTGGATATGTCAAGGTGAAACATTTCAGAGTAAAACTGGTCTAGCCTAGCATCTTGGGAAGCAAGGCTGGTTGCTGAAGGTCCGTCACTCAATCCTTTCTTAGGTGCCTATACTTTATTTAATAATACATGTCAACGTTTTGCTTAGAGGCATACAGACCCTTGGACTCCCAAGAAAGAACTGGTCTTATTTATCAGACAACTAAAATAAGCAAAGGCACTTGGTAGGTAGAGCCTGTCAGGTCACATGGCAAGGTGGTGTCCAGAGCCTTAATACTAAATACATCTTCGGCAAGTTGGGCTCGCTCTTCTCCCTGTATCTGGGTGCTATTACTTCCGAGTCAACTTCATCTTTGGGAAATGCTGCCAGATTTGCCCTTGGATTCTGTTTTGGCTTGGGTTGAAACTGCCTTATTTTGACTGACCCCTGAAAAAAGAAACTATATATGTGAAACCATCAATTTACAGTCTACAATCACTGAGTTGTTTTCGTGGTTTTTGTTTTTGTTTTTCTTAGGAGGTGCCCAGACATGGGTGACTGGAGCGCCTTGGGGAAGCTGCTGGACAAGGTCCAAGCCTACTCCACGGCCGGAGGGAAGGTGTGGCTGTCGGTGCTCTTCATTTTCAGAATCCTGCTCCTGGGGACAGCGGTTGAGTCAGCTTGGGGTGATGAACAGTCTGCCTTTCGCTGTAACACTCAACAACCCGGTTGTGAAA
